# Chitosan–Platelet-Rich Plasma Implants Improve Rotator Cuff Repair in a Large Animal Model: Pivotal Study

**DOI:** 10.3390/pharmaceutics13111955

**Published:** 2021-11-18

**Authors:** Anik Chevrier, Mark B. Hurtig, Marc Lavertu

**Affiliations:** 1 Chemical Engineering Department, Polytechnique Montreal, 2900 Boul. Édouard-Montpetit, Montreal, QC H3T 1J4, Canada; anik.chevrier@polymtl.ca; 2 Department of Clinical Studies, University of Guelph, Guelph, ON N1G 2W1, Canada; mark.hurtig@gmail.com; 3 Biomedical Engineering Institute, Polytechnique Montreal, 2900 Boul, Édouard-Montpetit, Montreal, QC H3T 1J4, Canada

**Keywords:** chitosan, platelet-rich plasma, rotator cuff repair, sheep model

## Abstract

The purpose of this study was to assess the safety and efficacy of chitosan–platelet-rich plasma (PRP) hybrid implants used as an adjunct to surgical rotator cuff repair in a pivotal Good Laboratory Practice (GLP)-compliant study. The infraspinatus tendon was transected in 48 skeletally mature ewes and repaired with a transosseous-equivalent (TOE) technique. In the two treatment groups, a chitosan–PRP solution was injected at the footprint between the tendon and the bone and on top of the repaired site (2 mL or 3 mL doses, *n* = 12 per group). To further assess chitosan safety, a chitosan–water solution was injected at the same sites (3 mL, *n* = 12). Outcome measures included Magnetic Resonance Imaging (MRI) assessment and clinical pathology at 3 months and 6 months and histopathology at 6 months. The tendon gap was decreased at 3 months on MRI images and certain histopathological features were improved at 6 months by chitosan–PRP treatment compared to controls. The group treated with chitosan–water was not different from controls. Chitosan–PRP treatment induced no negative effects in the sheep, which suggests high safety. This study provides further evidence on the safety and efficacy of chitosan–PRP for rotator cuff repair augmentation, which could eventually be used in a clinical setting.

## 1. Introduction

The rotator cuff plays an essential role in maintaining shoulder function and stability by pressing the humeral head on the glenoid and transmitting and absorbing forces. Epidemiology studies have revealed that rotator cuff tears are common in the general population, especially in older people (overall prevalence of ~20%; prevalence of ~40% in people 70–80 years) [1,2]. Once the rotator cuff tears, structural changes including fatty accumulation in the muscle, loss of muscle volume and muscle retraction occur, which can cause shoulder dysfunction and pain in some patients, although some remain asymptomatic [1,2]. The current standard-of-care involves surgical reattachment of torn tendons with suture anchors, but failures occur frequently. Re-tearing is more likely in patients that had initial larger tears [3] (~10% in tears < 2 cm versus ~50% in tears > 6 cm) and in older patients [4]. Basic studies have shown that tendon–bone healing occurs through the formation of fibrovascular scar tissue that is structurally different than the native tendon–bone insertion site, which might explain why some repairs fail. Regeneration of the stratified tendon–bone interface through repair augmentation strategies might decrease failures.

Platelet-rich plasma (PRP) has been used as a strategy to augment surgical rotator cuff repair, but results have been inconsistent so far, with some studies reporting small-to-moderate beneficial effects [5] and other studies reporting no effect [6], or even a negative effect [7]. These inconsistencies might result from the poor stability and residency of PRP in vivo. A well-known property of PRP is that it retracts to a small proportion of its original volume post-coagulation in a process that is mediated by platelets and that it is susceptible to lysis in physiological environments [8,9]. We hypothesized that PRP bioactivity in vivo could be increased by increasing its stability and its residency.

To achieve this goal, we developed freeze-dried formulations of chitosan that also contain trehalose as a lyoprotectant and calcium chloride for PRP solidification [10,11,12]. Chitosan is a cationic biopolymer that is derived from chitin that has been shown to be beneficial to healing [13,14,15], including the healing of acute rotator cuff tears in a rat model [16]. These freeze-dried formulations were previously characterized in vitro [10,11,12] and were shown to dissolve rapidly in PRP to make a viscous, adhesive chitosan–PRP solution that solidifies to a gel-like state and allows the release of platelet-derived growth factors. Solid chitosan–PRP implants resist platelet-mediated retraction, are resident for ~6 weeks subcutaneously in vivo and induce cell recruitment, tissue synthesis and neo-vascularization, while PRP on its own retracts significantly, rapidly degrades and shows very little signs of bioactivity.

In a transosseous suturing rotator cuff repair model in the rabbit, we showed that chitosan–PRP implants are resident at the site of injection, and that they improve supraspinatus attachment to the humeral head through increased remodeling at the tendon–bone interface and suppress heterotopic ossification in the tendon tissue [17]. In feasibility and pilot studies using a sheep rotator cuff repair model, we then showed that chitosan–PRP implants used in conjunction with suture anchors improved infraspinatus tendon repair compared to suture anchors alone, as assessed by MRI and histopathology [18,19]. The tendon gap, which is the length measurement of abnormally hyperintense tissue attached to the humeral head, was decreased by treatment compared to controls at 3 months. Histological scores for tendon structural organization and structural appearance of the enthesis were improved by treatment compared to controls at 3 months.

As a follow-up to the previously described pilot sheep study [19], the current Good Laboratory Practice (GLP)-compliant pivotal study examined the effect of chitosan–PRP on rotator cuff repair using the same model, but with a larger number of sheep in each group and with a longer repair period of 6 months. Our starting hypothesis was that using chitosan–PRP as an adjunct to surgical rotator cuff repair would lead to improvements compared to surgical repair alone. We also examined the effect of implant dose on efficacy and safety.

## 2. Materials and Methods

### 2.1. Preparation of the Freeze-Dried Chitosan Formulation

A current Good Manufacturing Practice (cGMP)-compliant facility (KABS Laboratories, Saint-Hubert, Québec, Canada) manufactured the ready-to-use freeze-dried chitosan formulation. The manufacturing process consists of the following steps: (1) Deacetylation of high-quality chitosan in sodium hydroxide followed by depolymerization in nitrous acid to obtain custom chitosan of the desired degree of deacetylation and molar mass; (2) dissolution of chitosan in mild hydrochloric acid and mixing with trehalose and calcium chloride; (3) sterilization by filtration, aliquoting into single-use vials and lyophilization of the chitosan formulation. Each vial (Lot X18M02E0) contained 50 mg chitosan (number average molar mass of 37 kDa and a degree of deacetylation of 84.6%), 50 mg trehalose and 23 mg calcium chloride. Each vial was solubilized in either autologous PRP or in sterile water for injection prior to application as further described below.

### 2.2. Rotator Cuff Tear Model and Study Design

The study included 48 healthy skeletally mature Rideau Arcott ewes (47–71 kg), which were group-housed in a room with concrete floors covered by wood chip bedding and a rubber mat. The animals had environmental acclimation for at least 2 weeks at the testing site. On the day of surgery, the animals were first anesthetized with diazepam (0.3 mg/kg) and ketamine (5 mg/kg), intubated, and then mechanically ventilated with isoflurane in O_2_. To guarantee the appropriate level of analgesia during and after the surgical procedure, Buprenorphine Slow Release (0.1 mg/kg, subcutaneous SC) and Rimadyl (4 mg/kg, SC) were administered during preparation for surgery. To prevent intraoperative infection, the antibiotic Cefazolin, 25 mg/kg, was given intravenously (IV). Bupivacaine 0.25% (up to 5 mL/surgical site) was infiltrated into the subcutaneous tissue to achieve local anesthesia and manage pain after surgery. Duplocillin (procaine and benzathine penicillin 1 mL/20 kg, intramuscular IM) and Excede (ceftiofur 5 mg/kg, IM) were given during recovery from anesthesia. Rimadyl (carprofen), 4 mg/kg, was administered once per day on days 2 to 4. Isoflurane level, blood oxygen saturation, heart rate and temperature were regularly monitored and manually recorded. Electrocardiograms were monitored but not recorded or retained. No complications occurred during surgery.

**Figure 1:**
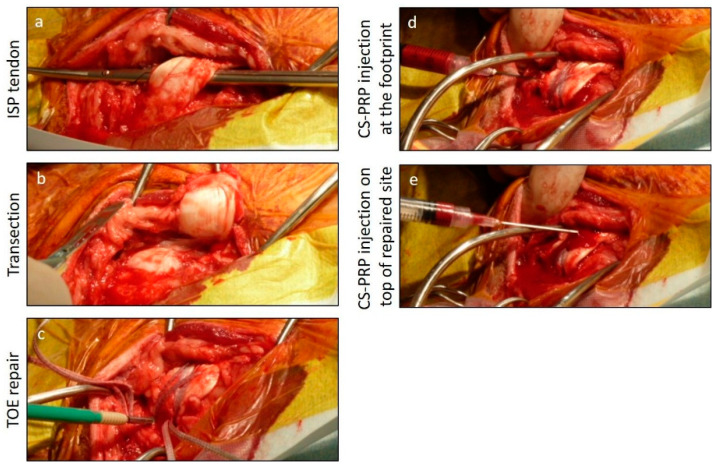
Rotator cuff model. The infraspinatus (ISP) tendon (**a**) was transected (**b**) and immediately repaired using a transosseous-equivalent (TOE) technique (**c**). Half the volume of the chitosan–PRP dose was injected at the footprint between the tendon and the bone (**d**) and half the volume was injected on top of the repaired site (**e**). CS: Chitosan; PRP: Platelet-rich plasma.

A unilateral infraspinatus tendon defect was created using extensive skin preparation, the application of an iodine-impregnated adhesive drape, sterile disposable drapes and a strict aseptic technique throughout the procedure (Figure 1). The subcutaneous colli muscle and brachial fascia were divided in line with the incision parallel to the spine of the scapula and curving over the lateral tuberosity. The plane of dissection was established along the cranial border of the acromial head of the deltoid muscle. The insertion of the infraspinatus tendon was isolated and elevated with a hemostat. The infraspinatus tendon was transected at its insertion on the lateral tubercle of the humerus, exposing the underlying fibrocartilage and periosteal border, which was used as a landmark for the reconstruction. The surface of the tuberosity was roughened with the use of a small hand rasp, prior to anchor insertion. The aim was to lightly remove soft tissue, preserve the cortical bone and avoid aggressive decortication of the bone. The tendon was slightly abraded.

The infraspinatus tendon was repaired directly to the tuberosity with the use of four suture anchors in a transosseous-equivalent (TOE) configuration (Figure 1). The Arthrex SwiveLock drill and tap set were used to create the lateral holes approximately 5 mm proximal to the junction between the fibrocartilage and periosteum of the lateral tubercle of the humerus. The two proximal anchors (Arthrex 4.75 mm PEEK SwiveLock Product N° AR-2324PSCL) were threaded with the sutures (Arthrex Fiber Tape 2 mm Product N° AR-7237), then the sutures were passed through the proximal aspect of the infraspinatus tendon using the Arthrex FastPass Scorpion device. The second distal row of anchors was inserted approximately 1.5 cm distal to the proximal row just distal to the footprint of the infraspinatus tendon with the alternate anterior and posterior sutures from the proximal anchors to create the TOE configuration.

Animals were randomly picked in their housing enclosure by the animal facility technicians and assigned to one of four groups in a pre-defined sequence alternating between right and left shoulders. Data from our previous sheep study [19] were used to calculate the sample size for this pivotal study setting the significance level alpha (α) at 0.05 and Power (1-β) at 0.8 [20]. The study design is described in Table 1 and included two test groups treated with chitosan–PRP (Groups 1 and 2), one chitosan safety group (Group 3) and one standard-of-care control group (Group 4), all with *n* = 12 per group. For Groups 1 and 2, autologous leukocyte-rich PRP was isolated from each ewe immediately prior to surgery using a Harvest SmartPrep 2 centrifuge and associated kits (PC-60 PRP Procedure pack Product N° 51401). The resulting PRP had average platelet content of 1380 × 10^9^/L (4.4X that of whole blood) and contained leukocytes (20.0 × 10^9^/L) and erythrocytes (9.0 × 10^12^/L). For Groups 1 and 2, each freeze-dried chitosan vial was solubilized with 5 mL autologous leukocyte-rich PRP and mixed by hand vigorously for 10 s prior to application using a syringe equipped with an 18-gauge needle. For Group 1, 1 mL of chitosan–PRP was applied at the footprint between the tendon and the bone and 1 mL was applied on top of the repaired site. For Group 2, 1.5 mL of chitosan–PRP was applied at the footprint between the tendon and the bone and 1.5 mL was applied on top of the repaired site. Using the complete blood count analysis results, Group 1 theoretically received, on average, 2.6 × 10^9^ platelets and Group 2 received, on average, 4.4 × 10^9^ platelets at the repaired sites. Group 3 was a chitosan safety group in which the chitosan vials were solubilized with 5 mL sterile water for injection and applied at the footprint between the tendon and the bone (1.5 mL) and on top of the repaired site (1.5 mL). For Groups 1, 2 and 3, the shoulders were closed exactly 5 min after application of the chitosan–PRP or chitosan–water solutions. Group 4 was the TOE standard-of-care repair, without any chitosan or PRP applied.

Following the surgery, the animals were returned to their enclosure and received assistance to stand without struggling. The sheep were then placed in smaller enclosures in order to limit any brisk movements, but the animals were allowed to move freely within the enclosures. Animals were transferred to a long-term housing facility after 28 post-operative days, where they were group-housed on a concrete, anti-slip flooring covered with wood chip bedding. The animals had access to hay, complete ration pellets and water.

### 2.3. Magnetic Resonance Imaging (Mri) Imaging and Scoring

MRI imaging was performed at 3 and 6 post-operative months and sheep were kept under general anesthesia for scanning. Imaging was carried out in a clinical 1.5-T superconducting magnet with a coil-Flex Large (Siemens Magnetom Sonata Syngo 1.5 Tesla). The Syngo MR A30 software was used to acquire oblique axial images through the long axis of the rotator cuff tendon. Parameters for the proton density fast spin echo sequence were repetition time (TR) of 3600 ms, echo time (TE) of 28 ms; 15 cm^2^ field of view with a matrix of 256 × 256 with 512 × 512 interpolation; slice thickness of 2.1 mm; receiver bandwidth of 130 Hz/px; 3 repetitions. Parameters for the inversion recovery series were echo time (TE) of 28 ms; 15 cm^2^ field of view with a matrix of 256 × 256; slice thickness of 3 mm; receiver bandwidth of 145 Hz/px; 3 repetitions. Images were measured/scored independently as per Table 2 by experienced musculoskeletal radiologists (one MD radiologist, one veterinarian radiologist), who did not have knowledge of any treatment groups throughout the study. For tendon gap and tendon thickness measurements, the readers scrolled through all slices independently and decided which slice was the most representative of repair.

### 2.4. Histological Processing and Scoring

At 6 months post-treatment, the animals were euthanized by first inducing deep anesthesia and then administering a lethal injection of saturated potassium chloride IV as a rapid bolus. The 48 infraspinatus tendon/enthesis repaired samples and 4 randomly selected non-operated native sides were immersion-fixed in neutral buffered formalin, decalcified in formic acid, paraffin embedded and 5 µm sections were stained with Safranin O/Fast Green and Hematoxylin and Eosin. Histological sections were scored by a single reader (veterinarian pathologist) blinded to treatment as per Table 3, Table 4 and Table 5. The slides were transferred to a second veterinarian pathologist for a peer-review to ensure diagnostic consistency and scientific quality. Slides were scanned with a Nanozoomer RS, and NDP View software was used to export images.

### 2.5. Additional Outcome Measures Collected

Samples were collected from the heart (left ventricle with papillary muscle and a section of atrium; right ventricle with a section of atrium; septum with papillary muscle), kidneys (one transverse section of each kidney), liver (one section from the left lobe), lungs (one section from the left caudal and one section from the right middle lobes), axillary lymph nodes from the implanted side and spleen (one transverse section) and paraffin sections were stained with Hematoxylin and Eosin for descriptive histopathological evaluation. Animal monitoring and scoring for body weight, pain and lameness was performed twice daily during the first 10 days post-surgery and then monthly as per Table 6. Body weights were measured and clinical pathology samples (for hematology, coagulation, serum chemistry, urinalysis) were collected at baseline, 3 months and 6 months. In addition, synovial fluid smears were collected at 6 months for leukocyte differential analysis.

### 2.6. Statistical Analysis

Raw data were transferred to independent biostatisticians for analysis. All data collected were used, there were no exclusions. All analyses were performed using SAS Version 9.4 TS1M2 (SAS Institute, Cary, NC, USA). In the case where measurements were scored by 2 readers, reliability was determined by calculating intraclass correlation coefficients (ICCs) for continuous measures or Kappa statistics (Kappa) for categorical measurements, and agreement between readers was defined as poor (<0.00), slight (0.00–0.20), fair (0.20–0.40), moderate (0.40–0.60), substantial (0.60–0.80) or almost perfect (0.80–1.00). For continuous measures, between-group comparisons were performed using Wilcoxon rank-sum tests. Standardized effect sizes (Cohen’s d) were calculated and interpreted as representing small (Cohen’s d = 0.2), moderate (Cohen’s d = 0.5) or large (Cohen’s d = 0.8) differences between groups. For categorical measurements, Wilcoxon exact tests were used to compare experimental groups. To provide an analogous measure of Cohen’s d for categorical measures, a c-statistic was calculated. For the present study, c-statistics values of 0.6–0.7 were considered moderate evidence of an association, and c-statistics values > 0.7 were considered strong evidence of a relationship. Mixed effects modeling was also used to perform these comparisons while leveraging data from both readers, when available. Analyses were performed in an analogous manner to those described above, with a repeated animal effect included in the context of a linear (continuous measures) or cumulative logit (ordinal measures) mixed-effects regression model.

## 3. Results

Comparisons showed no statistically significant differences between the groups treated with either 2 mL or 3 mL of chitosan–PRP, therefore pooled results from those two groups are shown versus the standard-of-care control group for MRI outcomes at 3 and 6 months (Supplementary Tables S1–S4) and histopathology at 6 months (Supplementary Tables S5–S7). Comparisons showed no statistically significant differences between the group that was injected with the chitosan formulation solubilised in water without any PRP and the standard-of-care control group, and those results will not be further discussed.

### 3.1. Chitosan–PRP Implants Decreased Tendon Gap at 3 Months Post-Operative

**Figure 2:**
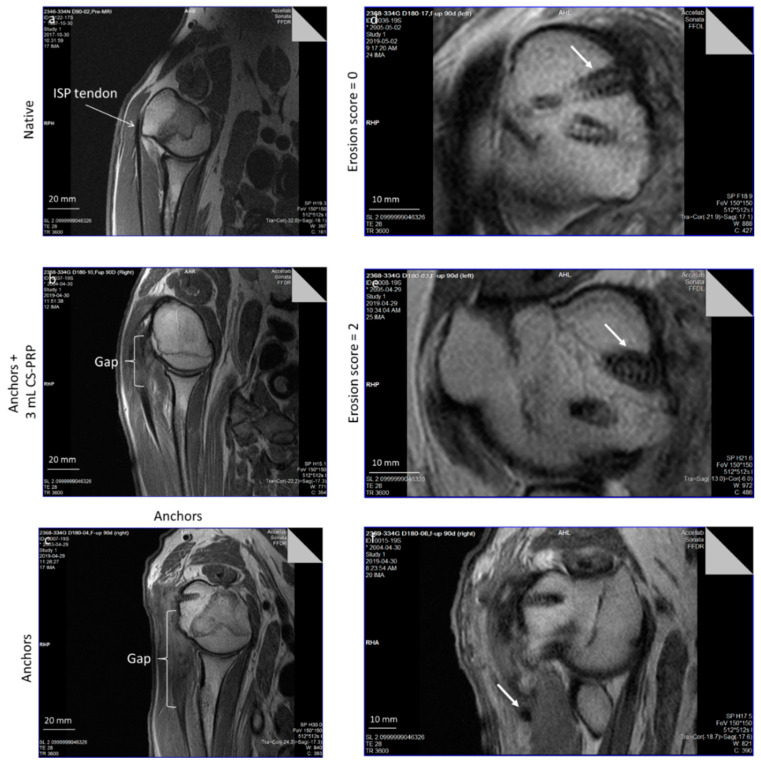
MRI images showing the tendon gap, which is not a “true” gap where tendon detached from bone but is the length of abnormally hyperintense tissue attached to the humeral head in panels (**b**,**c**) versus native hypointense infraspinatus tendon (ISP) in panel (**a**). Examples of shoulders that had erosion along the anchors scores of 0 in panel (**d**) and 2 in panel (**e**), with arrows pointing to anchors. Evidence of a retracted suture arrow in panel (**f**). CS: Chitosan, PRP: Platelet-rich plasma.

Certain MRI outcome measures showed moderate to substantial agreement between readers (tendon gap measurements with ICC = 0.464 and 0.666 at 3 and 6 months, respectively), while others showed only slight to fair reliability (erosion scores with Kappa = 0.275 and 0.133 at 3 and 6 months, respectively). Repaired tissues had structures on the MRI that were not identical to native (compare panels b and c versus a in Figure 2). Sutures that had retracted far away from the humeral head were observed in one animal from Group 1, one animal from Group 2, two animals from Group 3 and one animal from Group 4 (arrow in Figure 2f).

Among the continuous measurements, moderate to large differences were observed in the tendon gap at 3 months (effect size −0.39, *p* = 0.371 and effect size −0.78, *p* = 0.019 in reader-specific analyses), with smaller values measured in the chitosan–PRP treated groups when compared to standard-of-care controls (Supplementary Table S1). This decrease in the tendon gap at 3 months approached statistical significance (*p* = 0.060) in mixed-model analyses, with a difference of −5.21 mm (95% CI: −10.67, 0.24). At 6 months, the results for the tendon gap in mixed-model analyses supported a −4.89 mm (95% CI: −16.67, 6.89) lower value in the chitosan–PRP-treated groups, similar to the difference observed at 3 months, although it did not approach statistical significance due to wider confidence intervals (*p* = 0.405). Among categorical measurements, the group differences were generally modest in both reader-specific analyses (Supplementary Tables S3 and S4) and when combining both readers in mixed-model analyses. One exception was the erosion along the anchor scores for reader 2 at 6 months, which showed moderate but significant evidence of an association between chitosan–PRP treatment and erosion (c-stat 0.671, *p* = 0.023 Supplementary Table S4). Mixed-model analyses also showed a decreased likelihood of having smaller erosion scores among the chitosan–PRP-treated groups when compared to the standard-of-care control group at 6 months (*p* = 0.062).

### 3.2. Chitosan–PRP Implants Improved Some Histopathological Features at 6 Months

**Figure 3:**
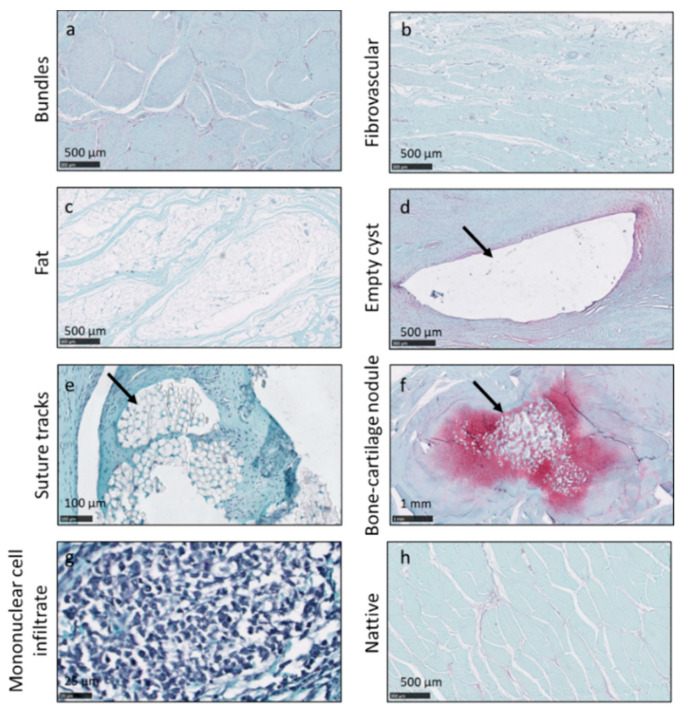
Histological findings in repaired tendons included tendon-like tissues that were organized in bundles (**a**), fibrovascular tissues (**b**), large fatty deposits (**c**), empty cysts (arrow in **d**), suture tracks (arrow in **e**), bone-cartilage nodules (arrow in **f**) and mononuclear inflammatory cell infiltrates (**g**). Panels a to e were from Group 1 animals. Panels (**f**,**g**) were from Group 4 animals. Native tendon is shown in panel (**h**).

None of the repaired tendons had a structure that was identical to the native tendon (Figure 3h). All repaired tendons were a mixture of tendon-like tissues that were organized in bundles (Figure 3a) and fibrovascular tissues (Figure 3b). Large fatty areas (Figure 3c) were observed in one tendon from Group 1 and two tendons from Group 4. Empty cysts (arrow in Figure 3d) were found in two tendons from Group 1 and two tendons from Group 3. Suture tracks were a common observation (arrow in Figure 3e). Bone–cartilage nodules (arrow in Figure 3f) were apparent in one tendon from Group 1, one tendon from Group 3 and two tendons from Group 4. Extensive mononuclear inflammatory cell infiltrates were observed in three tendons from Group 4 (Figure 3g).

**Figure 4:**
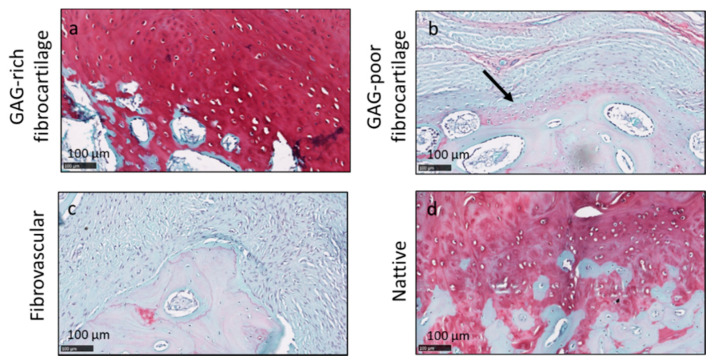
Histological findings in repaired entheses included tendon–bone interfaces that consisted of glycosaminoglycan (GAG)-rich fibrocartilage (**a**), GAG-poor fibrocartilage (arrow in **b**) and fibrovascular tissues (**c**). Panels (**a**–**c**) were from Group 1 animals. Native enthesis is shown in panel (**d**).

None of the repaired entheses had a structure identical to the native insertion site (Figure 4d). The repaired tendon–bone interfaces consisted of a mixture of glycosaminoglycan (GAG)-rich fibrocartilage (Figure 4a), GAG-poor fibrocartilage (Figure 4b) and fibrovascular tissues (Figure 4c). The calcified cartilage interface had partially reformed in most of the samples.

**Figure 5:**
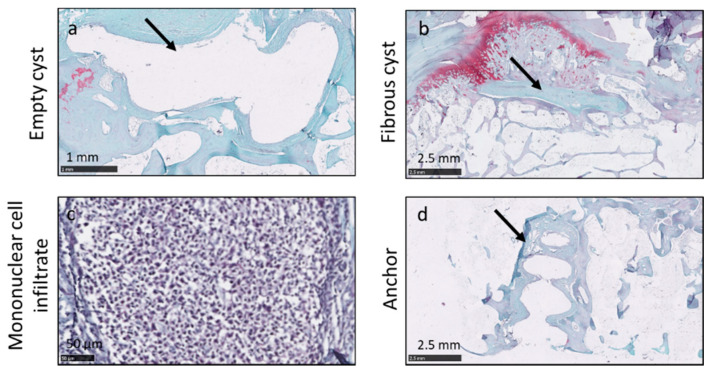
Histological findings in bone included empty (arrow in **a**) and fibrous (arrow in **b**) cysts, mononuclear inflammatory cell infiltrate (**c**) and anchor holes (arrow in **d**). All panels were from Group 4 animals.

Remodeling was apparent in the bone. An empty cyst (arrow in Figure 5a) was found in one sample from Group 4. Fibrous cysts (arrow in Figure 5b) were found in two samples from Group 2, one sample from Group 3 and one sample from Group 4. Extensive mononuclear inflammatory cell infiltrates (Figure 5c) were apparent in the bone of one sample from Group 4. Anchor holes were occasionally observed (arrow in Figure 5d).

**Figure 6:**
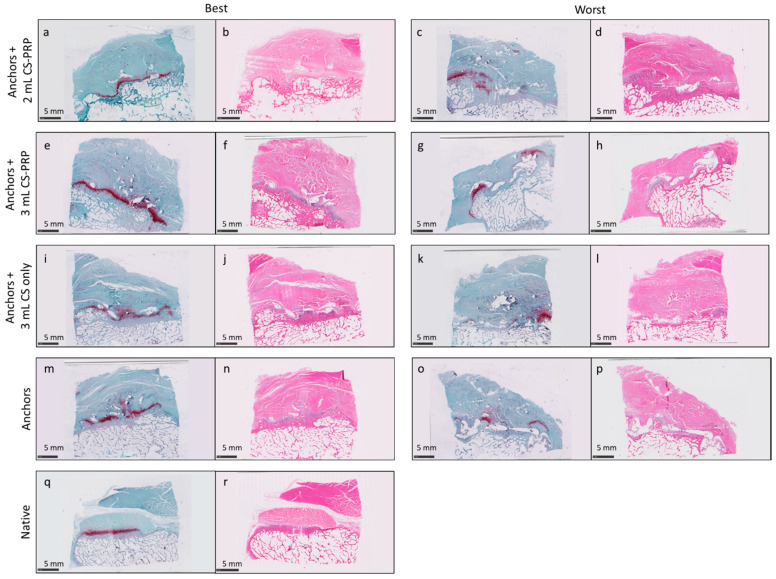
Safranin O/Fast Green and Hematoxylin/Eosin stained sections showing an example of the best and worst pan-enthesis site score for each treatment group in panels (**a**–**p**) and a native sample in panels (**q**,**r**).

Some histopathological features were improved by chitosan–PRP treatment. Data are consistent with less-severe cellularity (c-stat = 0.625, *p* = 0.031) and no evidence of inflammatory cells (c-stat = 0.708, *p* = 0.002) within the chitosan–PRP groups compared to standard-of-care controls (Supplementary Table S5). Specifically, 100% of samples in the chitosan–PRP-treated groups had mild cellularity, similar to native, whereas in the control samples, 75% had mild and 25% had moderate cellularity. Similarly, all chitosan–PRP-treated samples had no inflammatory cells observed in tendon tissue, compared to 41.7% of controls having minimal to moderate inflammatory cell scores. For ISP tendon insertion site measurements, the chitosan–PRP-treated groups were associated with an increase in the proportion of samples with no change or typical glycosaminoglycan staining compared to the controls (c-stat = 0.688, *p* = 0.071 Supplementary Table S6), particularly within the 3 mL dose group; 100% of the 3 mL dose group had typical glycosaminoglycan staining, whereas 75% were typical and 25% mild in the 2 mL dose group and 66.7% typical and 33.3% mild within controls. For ISP pan-enthesis measurements, chitosan–PRP-treated groups tended to have more complete healing of the enthesis site, characterized by a slightly higher magnitude of score (c-stat = 0.726, *p* = 0.019 Supplementary Table S7). One-third (*n* = 8/24) of chitosan–PRP-treated samples had complete healing with a smaller degree of remodeling, whereas none of the control samples fell within this category or better. In the treated groups, this corresponded microscopically to an overall better structured, well-organized enthesis site, with distinct, regular, well-organized tendon bundles, and fibrocartilage, generally combined with a lower score or magnitude of the bone remodeling. Figure 6 shows examples of the best and worst pan-enthesis site score for each treatment group.

### 3.3. No Treatment-Specific Effects on All Standard Safety Outcome Measures Were Detected

All animals survived to their scheduled sacrifice times. Pain was noted as being within normal limits for all sheep during the duration of the study and the wounds healed well despite suture line edema and small seromas. As expected across all groups, lameness and swelling were more often reported during the first few post-surgery weeks but diminished and disappeared in the long-term. The ewes had a stable weight or showed a mild increase from surgery to scheduled euthanasia. Regarding hematology, coagulation, serum chemistry and urinalysis data, groups were similar, and the vast majority of the data fell within the normal reference ranges. Excursions from the normal intervals were observed but they were deemed not clinically significant, and/or transient and/or marginal deviations caused by the experimental procedures. No differences were noted in macroscopic features, volume or cellularity of the synovial liquid when the groups were compared. There were no chitosan–PRP-related gross or microscopic findings in selected organs. Any animal’s health issues were not considered related to the administration of chitosan–PRP and were expected observations occasionally occurring in this experimental model post-surgery.

## 4. Discussion

MRI and histopathological data supported our starting hypothesis that surgical rotator cuff repair can be improved by the application of chitosan–PRP at the tendon–bone interface and on top of the repaired site. The tendon gap decreased at 3 months and certain histological features were improved at 6 months with chitosan–PRP treatment. Furthermore, both chitosan–PRP doses were well tolerated by the animals, which suggests high safety.

Rotator cuff disease may start as acute tendinopathy with progressive degeneration leading to a partial-thickness tear and eventually a complete tear. Rotator cuff tears can also result from an acute trauma, most frequently a fall onto an outstretched arm. Surgical repair techniques have evolved through the years in order to achieve a high initial fixation strength and maintain stability until tendon-to-bone healing can occur [22]. The infraspinatus tendon in sheep is similar in size, shape and microvasculature to the human supraspinatus tendon [23,24] and is large enough to allow the use of a standard-of-care repair technique. As in our previous pilot study [19], retracted sutures were observed in a few animals (5 out of 48), which suggests that the tendon had detached from the humeral head early post-operatively in those animals and that tissue ingrowth then filled the space between the retracted tendon and the humeral head, which was consistently observed in the case of transosseous suturing repair in a previous sheep rotator cuff repair study [21]. For the other animals in this study, it appears that the transosseous-equivalent repair technique provided enough fixation to allow first-intention healing of tendon-to-bone.

Even though modern surgical techniques provide strength to the initial repair, failures remain common and re-tearing is a significant problem, especially for older patients with larger initial tears [3,4]. One potential reason for failure is that the fibrovascular scar tissue that bridges the tendon–bone interface is weaker than the native enthesis and the forces transmitted during the rehabilitation process exceed the strength of the repair. Repair responses are robust in the sheep [25] and, as expected, no failures were observed at the end of this study. MRI and histopathological data showed that regeneration of a native-like enthesis was not achieved in any of the treatment groups. In contrast to our previous study [19], partial restoration of the tidemark was observed in most samples here, confirming that repair tissues undergo maturation between 3 months and 6 months in this model. As in our previous study [19], treatment with chitosan–PRP improved certain structural outcomes when compared to the standard-of-care. Complete healing of the enthesis site with a smaller degree of remodeling and more typical glycosaminoglycan staining confirm that a more organized and fibrocartilaginous interface was synthesized in the treated groups, which might translate into superior mechanical performance, although this was not assessed here and is a limitation of this study. As in the pilot study [19], the tendon gap was decreased by chitosan–PRP treatment 3 months post-operatively, which is indicative of a faster restoration of tissue with normal MRI signal.

The mechanisms by which repair is improved with chitosan–PRP treatment has not been fully elucidated. Phases of tendon healing typically include inflammation (lasting ~1 week), proliferation (lasting a few weeks) and remodeling (lasting several months) [26]. In this sheep model, at 1-day post-operative [19], chitosan–PRP has been shown to reside at the site of application at the enthesis and to adhere to infraspinatus tendon and muscle tissues at the repaired site due to the polycationic nature of solubilised chitosan [27]. Polymorphonuclear cells are recruited to those sites [19], where they contribute to chitosan degradation, so that the inflammatory phase of tendon repair will be prolonged until the chitosan is fully degraded at ~6 weeks. Implants composed of chitosan and whole blood have been shown to induce recruitment of stromal cells and differentiation of macrophages towards a pro-wound healing phenotype [28], both of which may have contributed to repair in the current study. Finally, the delivery and retention of autologous platelet-derived growth factors to the repaired site may be another mechanism by which chitosan–PRP contributes to repair. A moderate but significant increase in platelet-derived growth factor AB and epidermal growth factor release was quantified in vitro from chitosan–PRP when compared to PRP by itself [10]. In support of this notion, the application of the chitosan matrix solubilised in water without any PRP had no effect on repair in the current study. Another study limitation is that there was no group treated with PRP by itself, which might have allowed us to conclude that the combination of chitosan and PRP is essential for repair improvement. In the spirit of the 3Rs, and since autologous PRP safety has been well established, we elected to test the chitosan matrix solubilized in water and not include a PRP-only group in this study. Future research work will explore the modes of action of chitosan–PRP treatment and structure–function relationships using smaller animals that would be sacrificed at different time points post-surgery and performing non-destructive mechanical testing followed by detailed histological analyses.

One unexpected histological finding was the extensive mononuclear cell infiltrates that were found in the tendon and the bone tissues of four animals treated with the standard-of-care, and absent in all other animals. We currently have no explanation for this. It has been well established that immune cells play a role in degenerative tendinopathy and tendon healing following surgical repair [29,30]. Cases of inflammatory cell infiltration following rotator cuff repair with suture anchors and extracellular matrix patches have been reported [31,32,33], but it is unclear why this finding was restricted to animals in the control group in the current study. Another unexpected finding was the moderate but significant evidence of an association between chitosan–PRP treatment and scores for erosion along the anchors for one reader at 6 months. Of note, erosion scores were quite low (average score 0.6 for chitosan–PRP versus 0.4 for controls, out of a maximum score of 4), which suggests that any observed differences would be of questionable clinical significance. Reliability between readers was poor for this outcome measure and methods should be further refined for future studies. Drafting of a scoring method with detailed description and example figures would be helpful for this.

The model used here was an acute repair model where full-thickness tears were created and immediately repaired, which differs from the chronic nature of the disease often found in patients, and do not display muscle atrophy, stiffening, and fatty infiltration. Chronic rotator cuff repair models in the sheep have been described in the literature but they are challenging to execute [18], and often involve an osteotomy to detach the tendons [34,35,36], which does not accurately mimic the clinical situation. Another limitation of the sheep model is that it is difficult to immobilize them post-surgery, as is routinely done in people.

The results of this study were part of an investigational new drug (IND) submission to the U.S. FDA in order to initiate a Phase I/II clinical trial. Ahead of this trial, we used cadaveric shoulder specimens to test arthroscopic delivery of chitosan–PRP. We found that the chitosan–PRP mixture can be rendered very viscous and adherent by pre-incubating it for 30–45 min prior to delivery. The supraspinatus tendons of the cadaveric specimens were transected and arthroscopically repaired using a TOE technique. The inflow was closed, the arthroscopic fluid carefully aspirated via the outflow cannula and sterile surgical swabs were used to create a dry field at the surgically repaired site. Chitosan–PRP was then delivered at the footprint between the tendon and the bone and on top the repaired site and was found to adhere to the delivery site surfaces. In the cadaveric specimens, we applied 3 mL chitosan–PRP at the tendon–bone interface and 3 mL on top of the repaired site in order to account for differences in body mass, tendon volume and footprint area between sheep and humans. We feel confident that this arthroscopic delivery method will be appropriate for patients, and that standard rehabilitation protocols, which immobilize the limb for a few weeks post-surgery, will further contribute to the residency of chitosan–PRP. We believe that chitosan–PRP will improve rotator cuff healing following arthroscopic surgical repair, similar to what was seen here in the sheep model. The same chitosan–PRP formulation could eventually be used to aid in the repair of tendons or ligaments at other anatomical locations.

## 5. Conclusions

The tendon gap was decreased at 3 months on MRI images, which is indicative of a faster restoration of tissue with normal MRI signal, and some histological features were improved at 6 months with chitosan–PRP treatment in this large animal model. No treatment-specific effects were found on any of the safety outcome measures. This study provides further evidence on the safety and efficacy of chitosan–PRP for rotator cuff repair augmentation, which could eventually be used in a clinical setting.

## Figures and Tables

**Table 1:** Study design.

Group	Treatment	Dosage of Chitosan (mg)	Vehicle Used	Number of Animals	Time Point/Outcome Measure
1	Anchors + 2 mL CS-PRP	20	PRP	12	Baseline (Clinical pathology) 3 months (Clinical pathology + MRI) 6 months (Clinical pathology, MRI, Histopathology)
2	Anchors + 3 mL CS-PRP	30	PRP	12	
3	Anchors + 3 mL CS-water (Chitosan safety group)	30	Sterile water	12	
4	Anchors (Standard-of-care controls)	0	NA	12	

CS: Chitosan, PRP: Platelet-rich plasma; MRI: Magnetic resonance imaging.

**Table 2:** MRI parameters measured/scored.

Parameter Measured	Unit/Grade
Tendon gap ^1^	mm
Tendon thickness ^2^	mm
Tissue volume ^3^	cc
Presence of bursitis	None = 0; Mild = 1; Moderate = 2; Severe = 3
Synovial reaction	None = 0; Mild = 1; Moderate = 2; Severe = 3
Heterotopic bone formation	None = 0; Mild = 1; Moderate = 2; Severe = 3
Erosion of bone along the anchors	None = 0; At aperture only = 0.5; Along the entire anchor = 1 Note that each of 4 anchors were scored separately and then a sum was calculated Minimum score is 0 and maximum score is 4

^1^ Tendon gap is not a “true” gap where the tendon detached from the bone but was defined as the length of abnormally hyperintense tissue bridging the end of the native infraspinatus tendon and the bone at the humeral head [19,21] and was measured on a single slice that was most representative of the repair overall (Figure 2). ^2^ Tendon thickness was the thickest part of the tendon measured on a single slice that was most representative of the repair overall. ^3^ Tissue volume was measured by a single reader on the whole dataset.

**Table 3:** Histological scoring system for tendon tissues.

**Cellularity**	**Score**	**Tenocytes**	**Score**
None	0	Marked/Normal tenocyte cellularity	0
Minimal	1	Moderate (slight decrease tenocyte cellularity)	1
Mild	2	Mild	2
Moderate	3	Minimal	3
Marked	4	None	4
**Vascularity**	**Score**	**Glycosaminoglycan Expression**	**Score**
None	0	None	0
Minimal	1	Minimal	1
Mild	2	Mild	2
Moderate	3	Moderate	3
Marked	4	Marked	4
**Inflammatory Cells in Tendon Tissue**	**Score**	**Structural Organization**	**Score**
None (None observed and/or occasional isolated mononuclear cells)	0	Native tendon	0
Minimal (Few inflammatory cells)	1	Repair tissue mostly organized in bundles	1
Mild (More abundant inflammatory cells and/or locally extensive infiltration)	2	Repair tissue mostly aligned but not in bundles	2
Moderate (Larger locally extensive and/or more widespread inflammatory cell infiltration)	3	Repair tissue completely disorganized, but areas of tendon material can be identified	3
Marked (Dense inflammatory cell infiltration obscuring local architecture)	4	Complete loss of tendon architecture (minimal or no recognizable tendon material)	4

**Table 4:** Histological scoring system for enthesis tissues.

**Structural Appearance of the Enthesis**	**Score**
Native insertion with tidemark throughout	0
Insertion has continuity with bone ingrowth and fibrocartilage and tidemark partially present	1
Insertion has continuity with bone ingrowth and fibrocartilage cells but no tidemark	2
Insertion has continuity with fibrous tissue	3
Insertion has continuity with fat	4
No continuity	5
**Glycosaminoglycans (GAG) at Insertion Site**	**Score**
No change/typical appearance and/or subjective quantity of GAG staining	0
Slight (some GAG staining but faint), decreased compared to typical insertion site	1
None (complete absence of GAG staining)	2
**Bone Remodeling at Insertion Site**	**Score**
None	0
Minimal	1
Mild	2
Moderate	3
Marked	4

**Table 5:** Histological scoring system for pan-enthesis site.

**Pan-Enthesis Remodeling/Healing**	**Score**
No healing of enthesis site (i.e., complete absence of tendon reattachment)	0
Partial healing of enthesis site (absence of fibrocartilage and/or nearly complete replacement by fibrous enthesis) with disorganized tendon bundles	1
Complete healing of enthesis site with moderate remodeling (remodeling characterized by the presence of: variable thickness of fibrocartilage layer and/or bone remodeling and/or large nodular regions of fibrocartilage) with mostly organized tendon bundles and some fibrocartilage present	2
Complete healing of enthesis site with mild remodeling (variable thickness of fibrocartilage layer and/or bone remodeling and/or large nodular regions of fibrocartilage) with mostly organized tendon bundles and organized fibrocartilage present along most of the enthesis site	3
Complete healing of enthesis site with a smaller degree of remodeling (variable thickness of fibrocartilage layer and/or bone remodeling and/or large nodular regions of fibrocartilage) with well-organized tendon bundles associated with organized fibrocartilage mostly present along enthesis site	4
Complete healing of enthesis site with well-organized repair tissue; appears very similar/identical to native enthesis; recapitulates native anatomy	5
**Length of Insertion Site Present**	**Score**
No insertion site evidence on slide	0
Approximately <25% length of tissue on slide	1
Approximately 26–50% length of tissue on slide	2
Approximately 51–75% length of tissue on slide	3
Approximately >76% length of tissue on slide	4
**Quality/Consistency of Glycosaminoglycan (GAG) Staining of Cartilage/Fibrocartilage**	**Score**
Absence of GAG staining (no red color with Safranin-O)	0
Minimal GAG staining (decreased staining intensity)	1
Mild level of GAG staining (slightly decreased staining intensity)	2
Typical level of GAG staining +/− minimal staining of fibrous bundles associated with fibrocartilage	3
Excessive widespread GAG staining	4

**Table 6:** Scoring system for body weight, lameness and pain.

**Body Weight Scoring Stall Side**	**Score**
Emaciated	1
Thin	2
Normal	3
Overweight	4
Severely overweight	5
**Lameness Scoring Done Stall Side**	**Score**
Normal	0
Marginal intermittent lameness	1
Consistent mild lameness	2
Consistent moderate lameness	3
Consistent moderate lameness where the leg is used intermittently	4
Non weight bearing	5
**Pain Scoring Is Done Stall Side**	**Score**
Normal	0
Separation from group, quiet-alert-responsive, slightly increased respiratory rate	1
Separation from group, quiet-alert-responsive, slow to rise, reduced appetite, reduced rumen motility, increased respiratory rate	2
Separation from group, reluctant to rise when approached, no appetite, dull, teeth	3

## Data Availability

The data presented in this study are available on request from the corresponding author.

## Supplementary Materials

The following are available online at https://www.mdpi.com/article/10.3390/pharmaceutics13111955/s1, Table S1: Continuous MRI measurements at 3 months (chitosan–PRP versus controls), Table S2: Continuous MRI measurements at 6 months (chitosan–PRP versus controls), Table S3: Categorical MRI scores at 3 months (chitosan–PRP versus controls), Table S4: Categorical MRI scores at 6 months (chitosan–PRP versus controls), Table S5: Histological scores of tendon tissues at 6 months (chitosan–PRP versus controls), Table S6: Histological scores of enthesis tissues at 6 months (chitosan–PRP versus controls), Table S7: Histological scores of pan-enthesis site at 6 months (chitosan–PRP versus controls).
