# Supplementary material for: Chitosan–Platelet-Rich Plasma Implants Improve Rotator Cuff Repair in a Large Animal Model: Pivotal Study
Source: Pharmaceutics. 2021 Nov 18;13(11):1955. doi: 10.3390/pharmaceutics13111955 (PMC8622568; doi:10.3390/pharmaceutics13111955)
Supplement: Supplementary file 1 [file pharmaceutics-13-01955-s001.zip › pharmaceutics-1407904-supplementary.pdf]

pharmaceutics MDPI

Supplementary Materials: Chitosan-Platelet-Rich Plasma Implants Improve Rotator Cuff Repair in a Large Animal Model: Pivotal Study

Anik Chevrier, Mark B. Hurtig and Marc Lavertu

Table S1. Continuous MRI measurements at 3 months (Chitosan-PRP versus controls).

	Anchors (n = 12)		Anchors + CS-PRP (n = 24)		Anchors + CS-PRP vs Anchors	
**Reader 1**	**Mean**	**SD**	**Mean**	**SD**	**Effect size[1]**	**p[2]**
Tendon gap (mm)	44.3	10.9	40.5	8.9	−0.39	0.371
Tendon thickness (mm)	15.5	4.2	16.2	4.4	0.15	0.485
Tendon volume (cc)	13.1	7.7	12.7	5.3	−0.06	0.727
**Reader 2**	**Mean**	**SD**	**Mean**	**SD**	**Effect size[1]**	**p[2]**
Tendon gap (mm)	48.3	10.3	41.7	6.1	−0.78	0.019
Tendon thickness (mm)	13.5	2.6	14.4	4.0	0.29	0.777

[1] Cohen's d were calculated and interpreted as representing small (Cohen's d = 0.2), moderate (Cohen's d = 0.5) or large (Cohen's d = 0.8) differences between groups; [2] p-value from Wilcoxon test.

Table S2. Continuous MRI measurements at 6 months (Chitosan-PRP versus controls).

	Anchors (n = 12)		Anchors + CS-PRP (n = 24)		Anchors + CS-PRP vs Anchors	
**Reader 1**	**Mean**	**SD**	**Mean**	**SD**	**Effect size[1]**	**p[2]**
Tendon gap (mm)	31.6	18.0	25.4	17.1	−0.36	0.275
Tendon thickness (mm)	15.3	4.6	15.5	5.4	0.05	0.881
Tendon volume (cc)	9.2	5.5	11.0	6.0	0.31	0.281
**Reader 2**	**Mean**	**SD**	**Mean**	**SD**	**Effect size[1]**	**p[2]**
Tendon gap (mm)	24.6	21. 1	21.1	17.8	−0.18	0.562
Tendon thickness (mm)	12.1	4.6	11.9	4.2	−0.04	0.665

[1] Cohen's d were calculated and interpreted as representing small (Cohen's d = 0.2), moderate (Cohen's d = 0.5) or large (Cohen's d = 0.8) differences between groups; [2] p-value from Wilcoxon test.

Table S3. Categorical MRI scores at 3 months (Chitosan-PRP versus controls).

	Parameter	Anchors (n = 12)		Anchors + CS-PRP (n = 24)		Anchors + CS-PRP vs Anchors	
**Reader 1**	**Presence of Bursitis**	***n***	**%**	***n***	**%**	**c-stat[1]**	**p[2]**
	None (0)	5	41.7	9	37.5	0.505	1.000
	Mild (1)	6	50.0	15	62.5		
	Moderate (2)	1	8.3	0	0.0		
	Severe (3)	0	0.0	0	0.0		
	**Synovial Reaction**	***n***	**%**	***n***	**%**	**c-stat[1]**	**p[2]**
	None (0)	2	16.7	4	16.7	0.542	0.719
	Mild (1)	6	50.0	10	41.7		
	Moderate (2)	4	33.3	9	37.5		
	Severe (3)	0	0.0	1	4.2		
	**Heterotopic Bone Formation**	***n***	**%**	***n***	**%**	**c-stat[1]**	**p[2]**

	None (0)	4	33.3	6	25.0	0.528	0.781
	Mild (1)	4	33.3	7	29.2		
	Moderate (2)	0	0.0	6	25.0		
	Severe (3)	4	33.3	5	20.8		
	**Erosion Along the Anchors**	***n***	**%**	***n***	**%**	**c-stat[1]**	**p[2]**
	Sum = 0.0	3	25.0	7	29.2	0.521	0.829
	Sum = 0.5	7	58.3	9	37.5		
	Sum = 1.0	0	0.0	7	29.2		
	Sum = 1.5	0	0.0	0	0.0		
	Sum = 2.0	2	16.7	1	4.2		
	Sum = 2.5	0	0.0	0	0.0		
	Sum = 3.0	0	0.0	0	0.0		
	Sum = 3.5	0	0.0	0	0.0		
	Sum = 4.0	0	0.0	0	0.0		
**Reader 2**	**Presence of Bursitis**	***n***	**%**	***n***	**%**	**c-stat[1]**	**p[2]**
	None (0)	6	50.0	10	41.7	0.542	0.729
	Mild (1)	6	50.0	14	58.3		
	Moderate (2)	0	0.0	0	0.0		
	Severe (3)	0	0.0	0	0.0		
	**Synovial Reaction**	***n***	**%**	***n***	**%**	**c-stat[1]**	**p[2]**
	None (0)	2	16.7	3	12.5	0.608	0.321
	Mild (1)	10	83.3	16	66.7		
	Moderate (2)	0	0.0	5	20.8		
	Severe (3)	0	0.0	0	0.0		
	**Heterotopic Bone Formation**	***n***	**%**	***n***	**%**	**c-stat[1]**	**p[2]**
	None (0)	6	50.0	7	29.2	0.608	0.287
	Mild (1)	3	25.0	6	25.0		
	Moderate (2)	1	8.3	8	33.3		
	Severe (3)	2	16.7	3	12.5		
	**Erosion Along the Anchors**	***n***	**%**	***n***	**%**	**c-stat[1]**	**p[2]**
	Sum = 0.0	5	41.7	14	58.3	0.587	0.408
	Sum = 0.5	4	33.3	6	25.0		
	Sum = 1.0	3	25.0	4	16.7		
	Sum = 1.5	0	0.0	0	0.0		
	Sum = 2.0	0	0.0	0	0.0		
	Sum = 2.5	0	0.0	0	0.0		
	Sum = 3.0	0	0.0	0	0.0		
	Sum = 3.5	0	0.0	0	0.0		
	Sum = 4.0	0	0.0	0	0.0		

[1] c-statistic or Area Under the Receiver Operating Characteristic Curve (AUC). c-statistics from 0.6 – 0.7 were considered moderate evidence of an association, and c-statistics > 0.7 were considered strong evidence of a relationship; [2] p-value from Exact Wilcoxon test.

Table S4. Categorical MRI scores at 6 months (Chitosan-PRP versus controls).

	**Parameter**	**Anchors (*n* = 12)**		**Anchors + CS-PRP (*n* = 24)**		**Anchors + CS-PRP vs Anchors**	
**Reader 1**	**Presence of Bursitis**	***n***	**%**	***n***	**%**	**c-stat[1]**	**p[2]**
	None (0)	5	41.7	10	41.7	0.500	1.000
	Mild (1)	6	50.0	12	50.0		
	Moderate (2)	1	8.3	2	8.3		

	Severe (3)	0	0.0	0	0.0		
	**Synovial Reaction**	*n*	%	*n*	%	**c-stat[1]**	**p[2]**
	None (0)	4	33.3	2	8.3	0.599	0.323
	Mild (1)	5	41.7	16	66.7		
	Moderate (2)	3	25.0	5	20.8		
	Severe (3)	0	0.0	1	4.2		
	**Heterotopic Bone Formation**	*n*	%	*n*	%	**c-stat[1]**	**p[2]**
	None (0)	4	33.3	2	8.3	0.510	0.957
	Mild (1)	2	16.7	8	33.3		
	Moderate (2)	1	8.3	10	41.7		
	Severe (3)	5	41.7	4	16.7		
	**Erosion Along the Anchors**	*n*	%	*n*	%	**c-stat[1]**	**p[2]**
	Sum = 0.0	1	8.3	5	20.8	0.556	0.579
	Sum = 0.5	9	75.0	9	37.5		
	Sum = 1.0	1	98.3	7	29.2		
	Sum = 1.5	0	0.0	0	0.0		
	Sum = 2.0	1	8.3	3	12.5		
	Sum = 2.5	0	0.0	0	0.0		
	Sum = 3.0	0	0.0	0	0.0		
	Sum = 3.5	0	0.0	0	0.0		
	Sum = 4.0	0	0.0	0	0.0		
**Reader 2**	**Presence of Bursitis**	*n*	%	*n*	%	**c-stat[1]**	**p[2]**
	None (0)	5	41.7	14	58.3	0.559	0.633
	Mild (1)	7	58.3	8	33.3		
	Moderate (2)	0	0.0	2	8.3		
	Severe (3)	0	0.0	0	0.0		
	**Synovial reaction**	*n*	%	*n*	%	**c-stat[1]**	**p[2]**
	None (0)	1	8.3	7	29.2	0.585	0.348
	Mild (1)	11	91.7	16	66.7		
	Moderate (2)	0	0.0	1	4.2		
	Severe (3)	0	0.0	0	0.0		
	**Heterotopic Bone Formation**	*n*	%	*n*	%	**c-stat[1]**	**p[2]**
	None (0)	6	50.0	4	16.7	0.589	0.372
	Mild (1)	1	8.3	12	50.0		
	Moderate (2)	4	33.3	5	20.8		
	Severe (3)	1	8.3	3	12.5		
	**Erosion Along the Anchors**	*n*	%	*n*	%	**c-stat[1]**	**p[2]**
	Sum = 0.0	3	25.0	0	0.0	0.672	0.023
	Sum = 0.5	9	75.0	21	87.5		
	Sum = 1.0	0	0.0	3	12.5		
	Sum = 1.5	0	0.0	0	0.0		
	Sum = 2.0	0	0.0	0	0.0		
	Sum = 2.5	0	0.0	0	0.0		
	Sum = 3.0	0	0.0	0	0.0		
	Sum = 3.5	0	0.0	0	0.0		
	Sum = 4.0	0	0.0	0	0.0		

[1] c-statistic or Area Under the Receiver Operating Characteristic Curve (AUC). c-statistics from 0.6 – 0.7 were considered moderate evidence of an association, and c-statistics > 0.7 were considered strong evidence of a relationship; [2] p-value from Exact Wilcoxon test.

Table S5. Histological scores of tendon tissues (Chitosan-PRP versus controls).

Parameter	Anchors (n = 12)		Anchors + CS-PRP (n = 24)		Anchors + CS-PRP vs Anchors	
**Cellularity**	***n***	**%**	***n***	**%**	**c-stat[1]**	**p[2]**
None (0)	0	0.0	0	0.0	0.625	0.031
Minimal (1)	0	0.0	0	0.0		
Mild (2)[1]	9	75.0	24	100.0		
Moderate (3)	3	25.0	0	0.0		
Marked (4)	0	0.0	0	0.0		
**Tenocytes**	***n***	**%**	***n***	**%**	**c-stat[1]**	**p[2]**
Marked/Normal (0)	2	16.7	7	29.2	0.563	0.685
Moderate (1)	10	83.3	17	70.8		
Mild (2)	0	0.0	0	0.0		
Minimal (3)	0	0.0	0	0.0		
None (4)	0	0.0	0	0.0		
**Inflammatory Cells in Tendon Tissue**	***n***	**%**	***n***	**%**	**c-stat[1]**	**p[2]**
None (0)	7	58.3	24	100.0	0.708	0.002
Minimal (1)	2	16.7	0	0.0		
Mild (2)	1	8.3	0	0.0		
Moderate (3)	2	16.7	0	0.0		
Marked (4)	0	0.0	0	0.0		
**Vascularity**	***n***	**%**	**n**	**%**	**c-stat[1]**	**p[2]**
None (0)	0	0.0	0	0.0	0.521	1.000
Minimal (1)	9	75.0	17	70.8		
Mild (2)	3	25.0	7	29.2		
Moderate (3)	0	0.0	0	0.0		
Marked (4)	0	0.0	0	0.0		
**Structural Organization**	***n***	**%**	***n***	**%**	**c-stat[1]**	**p[2]**
Native tendon (0)	0	0.0	0	0.0	0.521	1.000
Repair tissue mostly organized in bundles (1)	2	16.7	5	20.8		
Repair tissue mostly aligned but not in bundles (2)	10	83.3	19	79.2		
Repair tissue completely disorganized, but areas of tendon material can be identified (3)	0	0.0	0	0.0		
Complete loss of tendon architecture (minimal or no recognizable tendon material) (4)	0	0.0	0	0.0		
**Glycosaminoglycan Expression**	***n***	**%**	***n***	**%**	**c-stat[1]**	**p[2]**
None (0)	0	0.0	0	0.0	0.509	0.954
Minimal (1)	2	16.7	0	0.0		
Mild (2)	1	8.3	8	33.3		
Moderate (3)	7	58.3	11	45.8		
Marked (4)	2	16.7	5	20.8		

[1] c-statistic or Area Under the Receiver Operating Characteristic Curve (AUC). c-statistics from 0.6 – 0.7 were considered moderate evidence of an association, and c-statistics > 0.7 were considered strong evidence of a relationship; [2] p-value from Exact Wilcoxon test.

Table S6. Histological scores of enthesis tissues (Chitosan-PRP versus controls).

Parameter	Anchors (*n* = 12)		Anchors + CS-PRP (*n* = 24)		Anchors + CS-PRP vs Anchors	
**Structural Appearance of the Enthesis**	*n*	%	*n*	%	**c-stat[1]**	**p[2]**
Native insertion with tidemark throughout (0)	0	0.0	0	0.0	0.521	1.000
Insertion has continuity with bone ingrowth and fibrocartilage and tidemark partially present (1)	10	83.3	19	79.2		
Insertion has continuity with bone ingrowth and fibrocartilage cells but no tidemark (2)	2	16.7	5	20.8		
Insertion has continuity with fibrous tissue (3)	0	0.0	0	0.0		
Insertion has continuity with fat (4)	0	0.0	0	0.0		
No continuity (5)	0	0.0	0	0.0		
**Glycosaminoglycans (GAG) at Insertion Site**	*n*	%	*n*	%	**c-stat[1]**	**p[2]**
No change/typical appearance and/or subjective quantity of GAG staining (0)	2	16.7	13	54.2	0.688	0.071
Slight (some GAG staining but faint), decreased compared to typical insertion site (1)	10	83.3	11	45.8		
None (complete absence of GAG staining) (2)	0	0.0	0	0.0		
**Bone Remodeling at Insertion Site**	*n*	%	*n*	%	**c-stat[1]**	**p[2]**
None (0)	0	0.0	0	0.0	0.561	0.583
Minimal (1)	0	0.0	1	4.2		
Mild (2)	1	8.3	6	25.0		
Moderate (3)	8	66.7	10	41.7		
Marked (4)	3	25.0	7	29.2		

[1] c-statistic or Area Under the Receiver Operating Characteristic Curve (AUC). c-statistics from 0.6 – 0.7 were considered moderate evidence of an association, and c-statistics > 0.7 were considered strong evidence of a relationship; [2] p-value from Exact Wilcoxon test.

Table S7. Histological scores of pan-enthesis site (Chitosan-PRP versus controls).

Parameter	Anchors (*n* = 12)		Anchors + CS-PRP (*n* = 24)		Anchors + CS-PRP vs Anchors	
**Pan-enthesis Remodeling/Healing**	*n*	%	*n*	%	**c-stat[1]**	**p[2]**
No healing of enthesis site (0)	0	0.0	0	0.0	0.726	0.019
Partial healing of enthesis site (1)	1	8.3	0	0.0		

Complete healing of enthesis site with moderate remodeling (2)	8	66.7	10	41.7		
Complete healing of enthesis site with mild remodeling (3)	3	25.0	6	25.0		
Complete healing of enthesis site with a smaller degree of remodeling (4)	0	0.0	8	33.3		
Complete healing of enthesis site with well-organized repair tissue; appears very similar/identical to native enthesis; recapitulates native anatomy (5)	0	0.0	0	0.0		
**Length of Insertion site Present**	*n*	%	*n*	%	**c-stat[1]**	**p[2]**
No insertion site evidence on slide (0)	0	0.0	0	0.0	0.521	1.000
Approximately <25% length of tissue on slide (1)	0	0.0	0	0.0		
Approximately 26-50% length of tissue on slide (2)	0	0.0	0	0.0		
Approximately 51-75% length of tissue on slide (3)	0	0.0	1	4.2		
Approximately >76% length of tissue on slide (4)	12	100.0	23	95.8		
**Quality/Consistency of Glycosaminoglycan (GAG) Staining of Cartilage/Fibrocartilage**	*n*	%	*n*	%	**c-stat[1]**	**p[2]**
Absence of GAG staining (no red color with Safranin-O) (0)	0	0.0	0	0.0	0.604	0.190
Minimal GAG staining (decreased staining intensity) (1)	0	0.0	0	0.0		
Mild level of GAG staining (slightly decreased staining intensity) (2)	4	33.3	3	12.5		
Typical level of GAG staining +/- minimal staining of fibrous bundles associated with fibrocartilage (3)	8	66.7	21	87.5		
Excessive widespread GAG staining (4)	0	0.0	0	0.0		

[1] c-statistic or Area Under the Receiver Operating Characteristic Curve (AUC). c-statistics from 0.6 – 0.7 were considered moderate evidence of an association, and c-statistics > 0.7 were considered strong evidence of a relationship; [2] p-value from Exact Wilcoxon test.
